# Marital Satisfaction and Psychological Well-Being in Older Adults Living with Health Difficulties

**DOI:** 10.1093/geroni/igaf122.3303

**Published:** 2025-12-31

**Authors:** Hyunjoo An, Minju Seong, Inhye Jung, Miseon Kang, Hyo Jung Lee

**Affiliations:** Yonsei University, Seoul, Republic of Korea; Yonsei University, Seoul, Republic of Korea; Yonsei University, Seoul, Republic of Korea; Symbiotic Life-TECH Institute, Seoul, Republic of Korea; Yonsei University, Seoul, Republic of Korea

## Abstract

Difficulties in performing everyday activities, commonly faced by older individuals, are closely linked to a lower level of psychological well-being, often being observed as an increased number of depressive symptoms. Marital relationships in later life have a significance due to various experiences of loss and long-term togetherness. The health of both spouses, including functional and emotional aspects, plays a critical role in shaping their own psychological well-being. This study explores the effect of spouse’s health on older individual’s psychological well-being, considering the moderating role of marital satisfaction. Using data from the 2022 Korean Longitudinal Study of Aging, we identified 3,820 older adults who were married, with information on spouse’s functional and emotional health. PROCESS Macro Model 1 was employed for the analysis. Compared to both spouses without functional health difficulties, marital satisfaction moderated the effect of a spouse’s psychological well-being on the individual’s psychological well-being when either spouse experienced such difficulties. Particularly when marital satisfaction was high, it served as a protective factor in this association. These findings indicate that older adults’ psychological well-being is affected by his or her spouse’s health status, especially when facing functional health difficulties. In addition, their positive spousal relationship satisfaction functions as a moderator to buffer this negative effect. These results contribute to an urgent need for better understanding the experiences of older individuals within marriage considering difficulties in health experienced by their spouses. It also suggests that a high level of satisfaction for their own marital relationship can improve older individuals’ psychological well-being.

